# Frailty identifies early and non-cardiac healthcare utilization after cardiovascular hospitalization

**DOI:** 10.3389/fcvm.2026.1806271

**Published:** 2026-05-29

**Authors:** Noel Rivas-González, María López, María José Castro, Belén Martín-Gil, Elsa Rodríguez-Gabella, Irene Alcoceba-Herrero, Mercedes Fernández-Castro, J. Alberto San Román

**Affiliations:** 1 Continuing Education Department, Valladolid University Clinical Hospital, Valladolid, Spain; 2 Valladolid Biosanitary Research Institute (IBIOVALL), Valladolid, Spain; 3 Faculty of Nursing, University of Valladolid, Valladolid, Spain; 4 GIR Research Group on Multidisciplinary Assessment and Intervention in Health Care and Sustainable Lifestyles, University of Valladolid, Valladolid, Spain; 5 Department of Nursing Care Information Systems, Valladolid University Clinical Hospital, Valladolid, Spain; 6 Research Support Unit, Valladolid University Clinical Hospital, Valladolid, Spain; 7 Cardiology Department, Valladolid University Clinical Hospital, Valladolid, Spain; 8 Biomedical Research Networking Centre on Cardiovascular Diseases (CIBERCV), Madrid, Spain

**Keywords:** aged, cardiovascular diseases, frailty, healthcare services utilization, hospitalization

## Abstract

**Background:**

Frailty is highly prevalent among older adults hospitalized with cardiovascular disease and is associated with adverse clinical outcomes. While its relationship with mortality and readmissions is documented, less is known about how frailty influences post-discharge healthcare utilization patterns. This study aimed to analyze the association between frailty and post-discharge healthcare utilization, defined as time to healthcare events, over 365 days in older adults hospitalized with cardiovascular disease.

**Methods:**

This prospective cohort study included patients aged ≥60 years hospitalized with cardiovascular disease between March 2022 and April 2024. Frailty was assessed using the FRAIL scale, alongside functional status (Barthel Index) and routinely collected biochemical markers. Outcomes included time to first cardiology consultation, non-cardiac consultation, emergency department visit, unplanned readmission, and death during 365 days of follow-up. Time-to-event analyses were performed using Kaplan–Meier, and Cox regression models (95% CI; *p* < 0.05).

**Results:**

A total of 130 patients were included (male: 68.46%; female: 31.54%; mean age: 72.98 ± 7.67 years). Frail patients (35.38%) had longer hospital stays and lower functional status. In univariate analysis, frailty was associated with earlier cardiology visits (HR: 1.57; *p* = 0.042), non-cardiac consultations (HR: 1.71; *p* = 0.012), and unplanned readmissions (HR: 2.11; *p* = 0.030). In multivariate models, frailty remained independently associated with earlier non-cardiac visits (HR: 1.47; *p* = 0.017), alongside LDL cholesterol levels. Hospital stay emerged as a consistent predictor across multiple outcomes. Mortality during follow-up was low (1.54%) and did not differ by frailty status.

**Conclusion:**

Frailty was associated with earlier post-discharge healthcare use, particularly outside cardiology. Integrating frailty screening with simple functional and biochemical indicators may help identify older adults at risk of increased post-discharge healthcare utilization and support post-discharge planning in ageing populations.

## 1 Introduction

From a public health perspective, identifying frail patients at risk of early and repeated use of health services following hospitalization for cardiovascular disease is essential for optimizing transitional care and resource allocation in ageing populations. Previous research has shown that frailty is associated with increased healthcare utilization, highlighting its relevance for healthcare planning and resource management (1).

Frailty, defined as a decline in physiological reserve and resilience, is a major determinant of outcomes in older adults, increasing vulnerability to stressors and risk of adverse healthcare outcomes and functional decline (2, 3). In cardiovascular disease (CVD), it is highly prevalent, affecting 25.8% of patients with heart failure and 32.0% with coronary syndromes (4–6).

CVDs remain the leading cause of death worldwide, but mortality is not the only relevant endpoint in ageing cardiovascular populations, as it poses an increasing challenge for healthcare systems. In the United States, annual costs for adults aged 65 years and older are projected to rise from USD 364.00 billion in 2020 to USD 1.17 trillion by 2050, with the sharpest increase among those aged 80 years and older (+371%) due to frailty and multimorbidity (7). Similar trends are observed in Europe, where CVDs accounted for over 3 million deaths in 2021—around 40% of female deaths and 35% of male deaths—and cost EUR 155.00 billion (11% of health spending) (8). In Spain, a country with one of the most rapidly ageing populations in Europe and the setting for the present study, CVDs cause ∼120,000 deaths and 592,000 admissions annually (9).

Frailty has been independently associated with poor prognosis in older adults with cardiovascular conditions (10). In addition to its association with adverse clinical outcomes, frailty has been linked to a greater need for post-discharge care and increased a higher likelihood of unplanned hospital readmissions (11). Unplanned hospital readmissions are also more frequent among frail patients with CVDs, reaching an incidence of 34.40% (*p* = 0.02) (11). Compared to robust individuals, frail patients have shown a significantly increased risk of readmission (HR = 2.32; 95% CI: 1.93–2.80; *p* < 0.001), as well as those in a pre-frail state (HR = 1.73; 95% CI: 1.06–2.82; *p* = 0.028) (4, 5). These patterns underscore the importance of examining broader post-discharge outcomes, including healthcare utilization beyond disease-specific endpoints in frail older adults.

Despite extensive evidence linking frailty to adverse outcomes, less attention has been paid to how and when healthcare services are used after hospital discharge, particularly those occurring outside cardiology services. These events represent an important marker of clinical instability and system-level burden in ageing populations with CVDs. Beyond cardiovascular-specific outcomes, frailty has also been independently associated with increased healthcare utilization during the year following assessment, largely driven by inpatient medical and post-acute services, regardless of surgical status. These findings support frailty as a marker of high-risk, high-cost trajectories highlight its relevance for improving the quality and value of care in older adults (1).

Frailty encompasses physical, functional, and metabolic components, complicating standardized care strategies (14, 15). The FRAIL scale assessing fatigue, resistance, ambulation, illness, and weight loss offers a practical screening option, particularly in resource-limited cardiology wards (16, 17). It predicts readmissions in NSTE-ACS and functional decline post-surgery (11, 18). Additional markers such as age, functional status (Barthel Index), hemoglobin, albumin, lipid profile parameters, and the presence of comorbidities have shown prognostic value in older patients (19–24).

Despite the growing number of prediction models, few have been validated for routine use in clinical practice. Recent work highlights the importance of developing practical, evidence-based approaches to frailty assessment (25). However, it remains unclear whether frailty is associated with early use of healthcare resources such as outpatient consultations, emergency department visits, or readmission. In this context, our study explores whether a brief frailty tool, combined with simple clinical indicators, can help anticipate healthcare needs after hospital discharge.

This study examines the association between frailty, measured with the FRAIL scale, and multiple post-discharge time-to-event outcomes, including outpatient visits, unplanned readmissions, and mortality, over 365 days in older adults with cardiovascular disease. By combining frailty screening with simple functional and biochemical indicators, the study aims to identify clinically feasible predictors of post-discharge healthcare utilization.

## 2 Materials and methods

### 2.1 Design, population, and sample

An observational cohort study was conducted in patients aged 60 years and over who were admitted to a cardiology unit at a tertiary-level hospital within the public healthcare network of Castile and León, Spain, between March 2022 and April 2024.

A threshold of 60 years was selected to capture early frailty transitions and functional decline, which may precede the conventional age threshold of 65 years in cardiovascular populations (2, 3)

Patients with a hospital stay of less than three days, including those admitted for short interventional procedures or low-complexity elective cases, were excluded to minimize potential bias in frailty assessment. Additionally, patients from outside the hospital's coverage area or with incomplete follow-up were not included.

Participants were recruited consecutively upon admission to the cardiology unit.

Frailty was assessed within the first 72 h of admission to capture patientś baseline vulnerability before significant clinical changes during hospitalization.

This manuscript was prepared following the STROBE (Strengthening the Reporting of Observational Studies in Epidemiology) guidelines (26).

#### 2.1.1 Sample size

A minimum of 28 frail and 57 non-frail patients was required to detect differences using Log-rank survival analysis (80% power, *p* < 0.05). This calculation assumed a readmission rate of 0.344 (11), a ratio of frail and robust group sizes of 0.47, a hazard ratio (HR) of at least 2.32 for unplanned hospital readmission, and a 5% loss to follow-up (5).

### 2.2 Variables

#### 2.2.1 Sociodemographic variables

The collected variables included age (in years), sex (male/female as recorded at birth), and the clinical diagnosis at admission (arrhythmias, coronary artery disease, heart failure, valvular disease, or endocarditis).

#### 2.2.2 Clinical variables

Clinical parameters included body mass index (BMI, kg/m^2^) measured using a calibrated scale and stadiometer, abdominal circumference (cm) measured using a milli-metric tape, degree of dependency measured using the Barthel Index (independent = 100 points; mild dependence = 91–99 points; moderate dependence = 61–90 points; The Barthel Index was categorized for descriptive purposes only) (27), presence of diabetes mellitus (Yes/No), length of hospital stay (days), previous readmissions (Yes/No), presence of dyspnea (Yes/No), and blood biomarkers: hemoglobin (g/dL), albumin (g/dL), total cholesterol (mg/dL), HDL cholesterol (mg/dL), LDL cholesterol (mg/dL) and, serum creatinine (mg/dL).

Blood biomarker parameters were included because they are routinely collected upon hospital admission for cardiology patients and may reflect the nutritional, metabolic, and renal vulnerability associated with frailty.

#### 2.2.3 Frailty identification

Frailty status was assessed using the FRAIL scale, which consists of five domains evaluated using five questions (16):

1. How much of the time during the past 4 weeks did you feel tired?
2. By yourself and not using aids, do you have any difficulty walking up 10 steps without resting?
3. By yourself and not using aids, do you have any difficulty walking several hundred yards?
4. For 11 illnesses, participants are asked, “Did a doctor ever tell you that you have [illness]?” 1 = Yes, 0 = No. The total illnesses (0–11) are recoded as 0–4 = 0 and 5–11 = 1. The illnesses include hypertension, diabetes, cancer (other than a minor skin cancer), chronic lung disease, heart attack, congestive heart failure, angina, asthma, arthritis, stroke and kidney disease.
5. How much do you weigh with your clothes on but without shoes? [current weight]' “One year ago in (MO, YR), how much did you weigh without your shoes and with your clothes on? [weight 1 year ago]” Percent weight change is computed as: [[weight 1 year ago –current weight]/[weight 1 year ago]] * 100. Percent change >5 (representing a 5% loss of weight) is scored as 1 and <5 as 0.

A Spanish-translated version of the FRAIL scale was used, aligned with the original structure. Patients were categorized into three groups: robust (no positive domains), pre-frail (one or two positive domains), and frail (three or more positive domains).

The Frail scale was selected because it is routinely integrated into our hospitaĺs electronic patient records, facilitating systematic frailty screening during hospitalization.

The internal consistency of the FRAIL scale in this cohort was acceptable for a brief clinical screening tool (Cronbach´s *α* = 0.687).

#### 2.2.4 Dependent variables

The following time-based outcomes were measured (in days): time to first follow-up cardiology visit, time to first medical visit for non-cardiac reasons, time to first emergency room visit, time to unplanned hospital readmission, and time to death.

Consultations were defined as outpatient medical visits recorded in the patient´s electronic record after hospital discharge. These included both scheduled follow-up visits and unscheduled outpatient consultations within the public healthcare system.

### 2.3 Procedures

One week before the study, nurses received a one-hour training session on administering the frailty scale to minimize inter-rate variability. This scale was included in the patient's electronic record (GACELA Care®) but was not used systematically in routine practice.

Patients were recruited consecutively after admission, and data were collected within the first three days after informed consent, ensuring measurements occurred before clinical deterioration. Nurses administered the FRAIL scale and took anthropometric measurements; remaining variables were obtained from medical records.

Post-discharge follow-up at one month, three months, six months, and one year used electronic records. Cases with incomplete follow-up (<5%) were excluded from time-to-event analysis.

### 2.4 Ethical considerations

Participant anonymity and data confidentiality were safeguarded using the Research Electronic Data Capture (REDCap) software (https://project-redcap.org/), in compliance with European Regulation 2016/679 of the European Parliament and Council of April 27, 2016, and Spanish Organic Law 3/2018 of December 5, on Personal Data Protection and Digital Rights Guarantee. The study was conducted by the Declaration of Helsinki and was approved by the Ethics Committee for Research with medicinal products (ECRmp) involving humans under the reference code PI-20-1612 on 23 January 2020. Informed consent was obtained from all subjects involved in the study.

### 2.5 Statistical analysis

The normality of the data was assessed using the Kolmogorov–Smirnov test, kurtosis, and skewness. Quantitative variables were presented as mean and standard deviation (SD), while categorical variables were expressed as frequency distributions. Quantitative variables were compared using Student's *t*-test when normality assumptions were met. When deviations form normality were identified, non-parametric tests (Mann–Whitney U) were used., Categorical variables were compared using Pearson's chi-squared test. The homogeneity of variance was verified using Levene's test.

Survival analysis was performed using Kaplan–Meier survival curves, with result sensitivity reinforced through the Mantel–Cox Log-rank test. To estimate the hazard rate (HR), univariate and multivariate Cox regression analyses were used. Additionally, a backward stepwise multivariate model was applied to refine the models and identify a parsimonious set of predictors associated with each outcome. Candidate variables were selected based on clinical relevance and prior evidence from the literature on frailty, functional status, and cardiovascular outcomes in older adults. The number of events per outcome was used to guide the specification of multivariable Cox models. Models were constructed separately for each outcome, and the number of predictors included was limited according to the events-per-variable principle (approximately ≥10 events per variable) to reduce the risk of overfitting. A 95% confidence interval and a significance level of *p* < 0.05 were applied.

Statistical analyses were conducted using IBM® SPSS® Statistics v.29 (SPSS, Inc., Chicago, IL, USA).

## 3 Results

**Figure 1:**
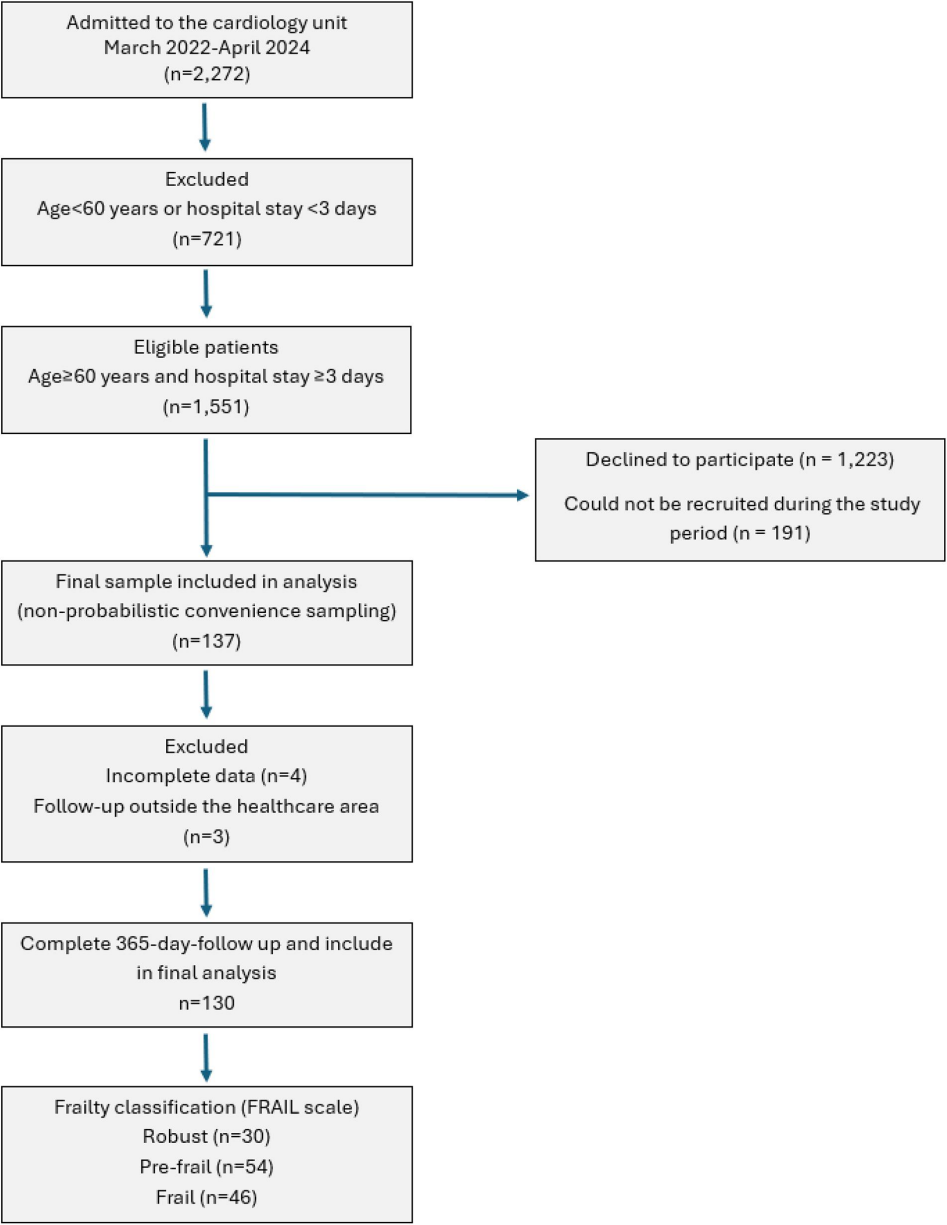
Flowchart of the participant selection process and classification according to frailty status.

Of the 137 eligible patients, four were excluded due to incomplete data, and three were excluded because they received care from other healthcare providers during the follow-up period, resulting in a final sample of 130 participants (Figure 1).

Of the final 130 participants, 68.46% were men and 31.54% were women; the mean age was 72.98 years (SD = 7.67), and frailty was identified in 35.38% of the patients. The mean hospital stay was 8.84 days (SD = 5.68), significantly longer in frail patients (*p* = 0.040). Functional status, measured by the Barthel Index, averaged 96.00 (SD = 8.39), with robust individuals scoring significantly higher than frail ones (*p* = 0.003). Nutritional status was similar across frailty groups in terms of BMI. However, frail patients showed lower mean albumin (3.73 ± 0.72 g/dL vs. 4.14 ± 1.50 g/dL) and hemoglobin (13.48 ± 2.26 g/dL vs. 14.13 ± 1.63 g/dL) compared with robust patients, alongside higher creatinine (1.33 ± 1.26 g/dL vs. 0.95 ± 0.22 g/dL) (Table 1).

**Table 1:** Description of the total sample by frailty status and sex.

*n* = 130 Variable	Total	Frail (*n* = 46)	Pre-frail (*n* = 54)	Robust (*n* = 30)
	Mean (SD)	Mean (SD)	Mean (SD)	Mean (SD)
Age (years)	72.98 (7.67)	75.22 (8.68)	71.70 (7.22)	71.87 (6.13)
Male *n* = 89	73.04 (7.39)	74.00 (9.00)	72.00 (7.00)	73.00 (5.00)
Female i = 41	72.85 (8.34)	77.00 (8.00)	71.00 (8.00)	70.00 (8.00)
BMI (kg/m^2^)	27.58 (4.38)	27.49 (4.75)	27.98 (4.54)	26.99 (3.45)
Male	27.55 (4,06)	27.80 (4.50)	27.30 (4.30)	27.60 (3.00)
Female	27.64 (5.05)	26.70 (5.40)	29.20 (4.80)	25.50 (4.20)
Abdominal Circumference (cm)	102.58 (13.14)	103.84 (12.81)	102.44 (14.84)	101.18 (10.35)
Male^a^	104.60 (10.98)	105.90 (11.70)	103.60 (11.90)	104.20 (8.30)
Female	98.41 (16.28)	98.60 (14.40)	100.50 (19.00)	93.00 (11.50)
Barthel Index	96.00 (8.38)	93.26 (9.79)	96.30 (8.64)	99.67^b^ (1.27)
Male^a^	97.08 (6.02)	95.15 (7.23)	97.21 (6.05)	99.77^b^ (1.07)
Female	93.66 (11.78)	88.46 (13.60)	94.75 (11.86)	99.38 (1.77)
Total Cholesterol (mg/dL)	159.39 (43.78)	154.41 (49.53)	162.15 (41.26)	162.07 (39.34)
Male	152.12 (44.50)	151.06 (50.59)	154.47 (45.29)	150.09 (34.09)
Female^a^	175.17 (38.11)	162.92 (47.61)	175.20 (30.04)	195.00 (35.16)
HDL (mg/dL)	44.99 (14.24)	43.24 (11.52)	45.98 (14.25)	45.90 (17.81)
Male	42.33 (12.01)	42.36 (11.81)	43.50 (12.74)	40.45 (11.39)
Female^a^	50.78 (16.91)	45.46 (10.76)	50.20 (15.96)	60.88 (23.98)
LDL (mg/dL)	90.85 (36.88)	86.85 (41.42)	94.04 (35.26)	91.23 (32.71)
Male	86.94 (37.91)	84.73 (42.55)	89.79 (39.24)	85.86 (28.65)
Female	99.32 (33.42)	92.23 (39.51)	101.25 (26.60)	106.00 (40.38)
Albumin (g/dL)	3.98 (1.12)	3.73 (0.72)	4.14 (1.50)	4.06 (0.71)
Male	4.05 (1.32)	3.77 (0.71)	4.27 (1.86)	4.12 (0.83)
Female	3.83 (0.52)	3.65 (0.77)	3.90 (0.39)	4.12 (0.83)
Hemoglobin (g/dL)	13.78 (1.93)	13.48 (2.26)	13.84 (1.77)	14.13 (1.63)
Male	14.13 (1.98)	13.80 (2.38)	14.26 (1.70)	14.40 (1.74)
Female	13.03 (1.60)	12.65 (1.76)	13.12 (1.70)	13.40 (1.04)
Creatinine (mg/dL)	1.15 (0.82)	1.33 (1.26)	1.10 (0.47)	0.95 (0.22)
Male^a^	1.22 (0.95)	1.41 (1.47)	1.18 (0.50)	1.00 (0.22)
Female	0.99 (0.39)	1.15 (0.44)	0.95 (0.41)	0.81 (0.17)
Length of Stay (days)	8.84 (5.68)	11.35^b^ (7.74)	9.70 (9.00)	6.90 (3.01)
Male	9.56 (6.19)	12.18 (8.75)	10.53 (5.56)	7.00 (3.30)
Female	7.22 (3.97)	9.23 (3.70)	8.30 (9.30)	6.63 (2.20)

a Total mean: Statistically significant differences between sexes (*p*-value < 0.05).

b Statistically significant differences between states of frailty (*p*-value < 0.05).

Coronary artery disease was the most common diagnosis (59.23%). Diabetes mellitus was more prevalent in frail individuals (43.48% vs. 13.33% in robust; *p* = 0.017). Readmission to cardiology occurred in 30.43% of frail patients vs. 6.67% of robust ones (*p* = 0.039).

Sex-based analyses showed greater abdominal circumference in men (104.60 ± 10.98 cm vs. 98.41 ± 16.28 cm; *p* = 0.001), but women with pre-frailty had a higher average BMI. Robust men showed a near-complete level of independence compared with frail men (*p* = 0.015). Frail women were more dependent than women in other groups and than frail men, though not significantly. Blood biomarker patterns varied: robust women showed higher total and HDL cholesterol; the lowest HDL values occurred in frail men, and the lowest hemoglobin in frail women. Hospital stays were longer for frail patients of both sexes, with the longest average observed in frail men (Table 1).

### 3.1 Time-to-event analysis of healthcare utilization

Kaplan–Meier estimates showed that frail patients had shorter times to all recorded events compared with robust and pre-frail participants. The time to first non-cardiology consultation differed significantly between groups (χ^2^ = 8.61; d.f. = 2; *p* = 0.014), and time to hospital readmission showed a non-significant trend (χ^2^ = 5.23; d.f. = 2; *p* = 0.073) (Table 2). Mortality was low, with two deaths (1.54%; 1 frail, 1 pre-frail) over follow-up. Due to the low number of events in certain subgroups, these time-to-event comparisons should be interpreted with caution.

**Table 2:** Distribution and association between time-to-event and frailty status.

Survival Outcome (Time-to-event: days)	Frailty Status			Mantel–Cox		
	Frail Mean (SD)	Pre-frail Mean (SD)	Robust Mean (SD)	*χ* ^2^	df	*p*-value
Time to first cardiology visit	153.98 (139.03)	205.07 (135.04)	215.03 (129.85)	4.25	2	0.119
Time to first non-cardiac visit	105.63 (126.32)	165.91 (157.17)	223.70 (158.62)	8.61	2	0.014*
Time to emergency care	210.39 (149.45)	255.33 (138.16)	257.57 (142.04)	2.29	2	0.193
Time to unplanned readmission	250.43 (149.58)	328.22 (98.61)	295.33 (132.05)	5.23	2	0.073
Death	363.00 (30.20)	358.24 (49.67)	365.00 (0.00)	0.62	2	0.734

* *p* < 0.05.

**Figure 2:**
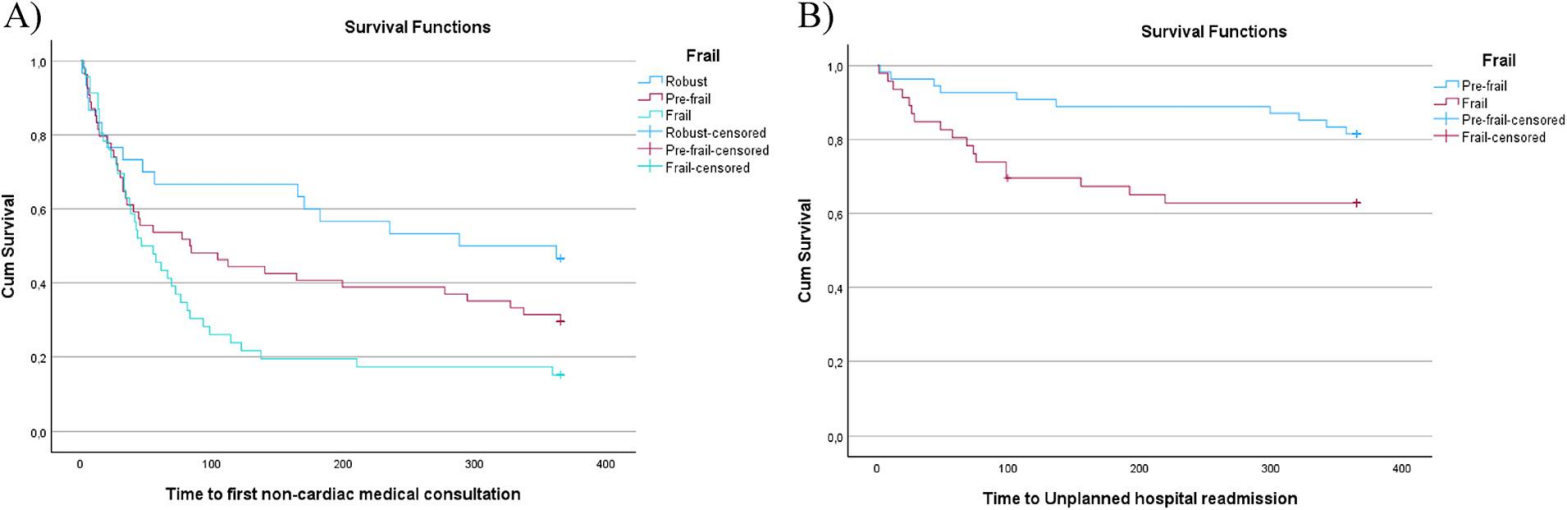
**(A)** Time-to-event (the first consultation for other reasons) across the three frailty groups. **(B)** Time-to-event (unplanned hospital readmission) amongra eritria. and frail patients.

At 359 days, 22.50% of frail patients had not required another consultation, compared with 26.90% for pre-frail at 365 days and 31.90% for robust (Figure 2A). For hospital readmissions, probabilities without readmission were 62.80% for frail patients at 219 days and 87.00% for pre-frail at 299 days (*p* = 0.025) (Figure 2B and Supplementary Table S1).

### 3.2 Univariate analysis

During the 365-day follow-up period, the number of observed events was as follows: 88 cardiology consultations, 95 non-cardiac consultations, 67 emergency department visits, and 35 unplanned hospital readmissions. These values correspond to the number of patients experiencing each event.

These event counts were considered when specifying multivariable models.

Cox regression indicated that frail patients required non-cardiology consultations sooner than robust and pre-frail combined (HR = 1.51; 95% CI: 1.14–2.00; *p* = 0.004). In the direct comparison between frail and robust patients, frailty was also associated with a shorter time to the first non-cardiological consultation (HR = 2.39; 95% CI: 1.32–4.33; *p* = 0.004). Grouping robust and pre-frail together showed shorter time-to-event for all outcomes in the frail group, though emergency visits were not statistically significant (Table 3).

**Table 3:** Univariate analysis of the association between frailty groups and time-to-event for the first cardiology consultation, consultation for other reasons, emergency care, or unplanned hospital readmission.

Frailty Comparison	Time-to-event (days) Outcome	B	df	*p*-value	HR	95% CI
Robust–Pre-frail–Frail	First cardiology consultation	0.26	1	0.073	1.30	0.98–1.74
	First non-cardiac consultation	0.41	1	0.004**	1.51	1.14–2.00
	Emergency care	0.27	1	0.107	1.31	0.94–1.83
	Unplanned hospital readmission	0.39	1	0.111	1.47	0.92–2.37
Frail vs. Pre-frail	First cardiology consultation	−0.42	1	0.086	0.66	0.41–1.06
	First non-cardiac consultation	−0.38	1	0.103	0.69	0.44–1.08
	Emergency care	−0.43	1	0.122	0.65	0.38–1.12
	Unplanned hospital readmission	−0.87	1	0.030*	0.42	0.19– 0.92
Frail vs. Robust	First cardiology consultation	0.47	1	0.100	1.61	0.91–2.82
	First non-cardiac consultation	0.87	1	0.004**	2.39	1.32– 4.33
	Emergency care	0.48	1	0.153	1.62	0.84–3.13
	Unplanned hospital readmission	0.54	1	0.228	1.72	0.71–4.15
Frail vs. Robust + Pre-frail	First cardiology consultation	0.45	1	0.042*	1.57	1.02–2.41
	First non-cardiac consultation	0.54	1	0.012*	1.71	1.13– 2.59
	Emergency care	0.45	1	0.073	1.56	0.96–2.55
	Unplanned hospital readmission	0.75	1	0.030*	2.11	1.08–4.14

B, Log-Hazard; HR, Hazard Ratio; CI, Confidence Interval.

* *p* < 0.05.

** *p* < 0.01.

Number of events: 88 cardiology consultations, 95 non-cardiac consultations, 67 emergency department visits, and 35 unplanned hospital readmissions.

### 3.3 Multivariate analysis by event

Backward stepwise Cox regression was used to derive the final multivariate models. To minimize overfitting, variable selection was based on statistical significance in univariate analyses and clinical relevance, ensuring an appropriate events-per-variable ratio.

The results of the final models are presented in Table 4.

**Table 4:** Univariate and multivariate analysis of clinical and sociodemographic variables regarding time-to-event for the first cardiology consultation, consultation for other reasons, emergency care, or unplanned hospital readmission.

Time-to-event Outcome	Variable	Univariate Analysis			Multivariate Analysis (final model)		
		*p*-value	HR	95% CI Lower–Upper	*p*-value	HR	95% CI Lower–Upper
First cardiology consultation *n* = 88	Frailty	0.073	1.30	0.98–1.74	–	–	–
	Age	0.069	1.03	0.99–1.05	0.039*	1.04	1.00–1.07
	Sex	0.646	1.11	0.71–1.74	–	–	–
	Diagnosis	0.207	1.19	0.91–1.57	–	–	–
	Previous readmission	0.545	1.20	0.67–2.16	–	–	–
	Diabetes mellitus	0.741	1.08	0.69–1.68	–	–	–
	Dyspnea	0.151	1.39	0.89–2.18	–	–	–
	Barthel Index	0.676	1.01	0.98–1.03	0.073	1.04	0.99–1.08
	Albumin	0.011**	0.64	0.46–0.91	0.074	0.70	0.48–1.03
	Hemoglobin	0.004**	0.86	0.77–0.95	0.065	0.89	0.79–1.01
	Creatinine	0.102	1.21	0.96–1.51	–	–	–
	Total cholesterol	0.264	0.99	0.99–1.00	–	–	–
	HDL cholesterol	0.592	1.00	0.99–1.02	–	–	–
	LDL cholesterol	0.496	0.99	0.99–1.00	–	–	–
	Body mass index (BMI)	0.535	0.99	0.94–1.03	–	–	–
	Abdominal circumference	0.307	0.99	0.98–1.01	–	–	–
	Length of hospital stay	<0.01**	1.08	1.04–1.12	0.001**	1.08	1.03–1.13
First consultation (non-cardiac) *n* = 95	Frailty	0.004**	1.51	1.14–2.00	0.017**	1.47	1.07–2.02
	Age	0.533	0.99	0.97–1.02	–	–	–
	Sex	0.834	1.05	0.68–1.63	–	–	–
	Diagnosis	0.604	1.07	0.83–1.38	–	–	–
	Previous readmission	0.203	1.42	0.83–2.44	–	–	–
	Diabetes mellitus	0.011*	1.72	1.13–2.61	–	–	–
	Dyspnea	0.523	1.16	0.74–1.81	–	–	–
	Barthel Index	0.317	0.99	0.97–1.01	–	–	–
	Albumin	0.273	0.85	0.63–1.14	–	–	–
	Hemoglobin	0.024*	0.89	0.79–0.98	–	–	–
	Creatinine	0.601	1.06	0.85–1.32	–	–	–
	Total cholesterol	0.066	0.99	0.99–1.00	–	–	–
	HDL cholesterol	0.556	0.99	0.98–1.01	–	–	–
	LDL cholesterol	0.034*	0.99	0.99–0.99	0.026*	0.99	0.98–0.99
	Body mass index (BMI)	0.207	1.03	0.98–1.08	–	–	–
	Abdominal circumference	0.577	1.00	0.99–1.02	–	–	–
	Length of hospital stay	0.155	1.03	0.99–1.07	–	–	–
Emergency care *n* = 67	Frailty	0.107	1.31	0.94–1.83	–	–	–
	Age	0.454	1.01	0.98–1.04	–	–	–
	Sex	0.404	1.24	0.75–2.06	–	–	–
	Diagnosis	0.883	1.02	0.76–1.38	–	–	–
	Previous readmission	0.072	1.75	0.95–3.21	–	–	–
	Diabetes mellitus	0.601	1.14	0.69–1.88	–	–	–
	Dyspnea	0.804	1.07	0.63–1.81	–	–	–
	Barthel Index	0.899	0.99	0.97–1.03	–	–	–
	Albumin	0.288	1.18	0.87–1.61	0.033*	1.33	1.02–1.72
	Hemoglobin	0.316	0.94	0.83–1.06	–	–	–
	Creatinine	0.329	0.79	0.49–1.27	–	–	–
	Total cholesterol	0.462	0.99	0.99–1.00	0.059	0.99	0.98–1.00
	HDL cholesterol	0.827	1.00	0.98–1.02	0.074	1.02	0.99–1.05
	LDL cholesterol	0.428	0.99	0.99–1.00	–	–	–
	Body mass index (BMI)	0.609	1.01	0.96–1.07	0.008**	1.20	1.05–1.38
	Abdominal circumference	0.727	0.99	0.98–1.01	0.012**	0.96	0.91–0.98
	Length of hospital stay	0.011*	1.06	1.01–1.10	0.001**	1.08	1.02–1.15
Unplanned hospital readmission *n* = 35	Frailty	0.111	1.47	0.91–2.37	–	–	–
	Age	0.707	0.99	0.95–1.04	–	–	–
	Sex	0.515	0.78	0.36–1.66	–	–	–
	Diagnosis	0.023*	1.55	1.06–2.27	–	–	–
	Previous readmission	0.196	1.73	0.75–3.98	–	–	–
	Diabetes mellitus	0.011*	2.39	1.22–4.69	0.029*	2.29	1.09–4.03
	Dyspnea	0.766	0.89	0.42–1.91	–	–	–
	Barthel Index	0.040*	0.97	0.94–0.99	–	–	–
	Albumin	0.255	1.13	0.92–1.38	–	–	–
	Hemoglobin	0.287	0.91	0.77–1.08	–	–	–
	Creatinine	0.679	1.07	0.78–1.46	–	–	–
	Total cholesterol	0.985	1.00	0.99–1.01	–	–	–
	HDL cholesterol	0.140	0.98	0.95–1.01	–	–	–
	LDL cholesterol	0.399	1.00	0.99–1.01	–	–	–
	Body mass index (BMI)	0.536	1.02	0.95–1.10	–	–	–
	Abdominal circumference	0.451	1.01	0.98–1.04	–	–	–
	Length of hospital stay	<0.001**	1.09	1.03–1.14	0.004**	1.08	1.03–1.14

*p < 0.05.

**p < 0.01.

Final model derived from backward stepwise Cox regression.

First cardiology consultation—Variables associated with the outcomes included age (HR = 1.04; *p* = 0.039) and hospital stay (HR = 1.08; *p* = 0.001). Barthel Index, albumin, and hemoglobin showed non-significant trends.

First non-cardiology consultation—Frailty was identified as an independent predictor (HR = 1.47; *p* = 0.017), along with LDL cholesterol (HR = 0.99; *p* = 0.026).

Emergency care—Patients who had longer hospital stays were 1.055 times more likely to require emergency care post-discharge for each additional day of hospitalization (*p* = 0.011). This relationship remained in the multivariate analysis (HR = 1.08; *p* = 0.001), along with higher albumin levels (HR = 1.33; *p* = 0.033), BMI (HR = 1.20, *p* = 0.008) and a smaller abdominal circumference (HR = 0.96; *p* = 0.012).

Unplanned hospital readmission—In the multivariate analysis, Diabetes Mellitus (HR = 2.29; *p* = 0.029) and length of hospital stay (HR = 1.08; *p* = 0.004) showed a statistically significant association as an independent predictive factors These results should be interpreted with caution due to the low number of events.

In multivariable models, sex was not independently associated with time-to-event outcomes, and no significant sex-by-frailty interactions were detected.

## 4 Discussion

The findings place frailty within a broader gerontological context, highlighting its relevance for understanding post-discharge care needs in older adults beyond disease-specific outcomes. In this cohort, frailty was mainly expressed as earlier use of non-cardiac healthcare services, rather than increased short-term mortality. We examined the association between frailty, as measured by the FRAIL scale, and time-to-event outcomes in older adults hospitalized with CVDs. Over 365 days of follow-up, frailty was associated with a less favorable clinical profile, longer hospital stays, greater functional impairment, and earlier healthcare utilization compared with pre-frail and robust patients. The prevalence observed (35.38%) is consistent with previously reported ranges for similar populations (4, 5, 11), and the high proportion of pre-frail patients highlights a potential window for early intervention (28).

Frail participants were older and had lower Barthel Index scores, supporting earlier findings that ageing and functional dependence tend to co-occur with frailty, although estimates for functional measures may vary across subgroups and study designs (29, 30). They also had higher creatinine levels. This pattern is consistent with renal impairment commonly associated with frailty and chronic inflammation (31), and a higher prevalence of diabetes mellitus and readmissions, in line with prior observations of increased comorbidity burden among frail individuals (32).

In time-to-event analysis, frailty was related to shorter time-to-event for non-cardiology consultations and showed a tendency towards earlier unplanned readmissions, patterns that have also been described elsewhere (4, 5). Frail patients often require a multidisciplinary approach to their treatment, and early referral to non-cardiology services may indicate unmet needs in this vulnerable population, reflecting potential difficulties in the continuity and coordination of care following hospital discharge. The reasons underlying outpatient consultations, emergency department visits, and readmissions were not systematically recorded in the dataset, preventing a detailed analysis of the clinical causes of healthcare utilization events. In Cox regression, frailty remained an independent predictor for earlier non-cardiology consultations, suggesting a broader vulnerability that may extend beyond cardiovascular disease (4, 18, 29). Conversely, higher Barthel Index scores appeared to be associated with a lower risk of unplanned readmission in univariate analysis, reinforcing the prognostic importance of functional status at discharge (30). In this population, the interpretation of frailty may serve as an indicator of broader vulnerability, rather than a predictor of isolated cardiovascular events.

Multivariate models suggested that hemoglobin, total cholesterol, HDL, LDL, and hospital stay length were associated with certain outcomes. Lower hemoglobin and hypoalbuminemia, often considered markers of nutritional vulnerability, have been associated with higher mortality risk (OR = 1.69; 95% CI = 1.47–2.91; *p* < 0.010) (20, 22, 33), supporting the relevance of nutritional status within comprehensive geriatric assessment. Anthropometric indicators such as BMI and abdominal circumference were also linked to emergency visits and readmissions, consistent with evidence that metabolic risk factors may be linked to both long-term and short-term outcomes (34, 35). Although some lipid-related variables reached statistical significance, their hazard ratios were close to 1.00, suggesting a limited clinical impact despite statistical association.

While sex was examined as a potential effect modifier, our exploratory cohort did not identify significant sex differences in the associations between frailty and time-to-event outcomes. However, women displayed greater functional dependency, echoing prior research on sex-based disparities in functional reserve and recovery capacity (12, 13).

Frailty was not retained as an independent predictor in all models, possibly due to overlap with comorbidity and metabolic variables. For cardiology follow-up, predictors included hospital stay length, and age were the main predictors (36, 37). For emergency care, BMI, abdominal circumference, and hospital stay appeared more relevant. For readmissions, diabetes mellitus, and hospital stay length were retained (38). Recent prospective data in heart failure populations are consistent with this interpretation. In a large outpatient cohort, this vulnerability has been shown to identify patients at higher risk of adverse clinical trajectories, particularly when coexisting with other chronic conditions (39). Complex predictive models should take all these factors into account, supporting the need to incorporate frailty assessment into routine cardiovascular care to improve prognostic stratification and individualized treatment and follow-up strategies in this patient population (40). This finding does not diminish the relevance of frailty, but rather supports its role as an integrative marker that captures the cumulative effect of comorbidity and overall physiological vulnerability. In this context, frailty could be interpreted as a multidimensional construct reflecting global risk rather than as an isolated independent predictor. In older and frail populations, the prognostic role of traditional cardiovascular risk factors may be modified, reflecting complex interactions between comorbidity, nutritional status, and physiological reserve. This may partly explain the heterogeneous and sometimes counterintuitive associations observed in this study.

The low mortality rate may reflect the inclusion of more clinically stable patients, which could limit the ability to detect associations with mortality; furthermore, it significantly reduces statistical power, preventing robust conclusions regarding mortality outcomes. Nevertheless, within this context, the FRAIL scale, though simple, offers a practical option in settings where discharge planning and transitional care for older adults require rapid and feasible assessment, especially when comprehensive geriatric assessment is not feasible. While our study did not compare multiple screening tools, recent evidence suggests that tools with higher specificity may be handy for ruling out non-frail patients and avoiding unnecessary allocation of preventive resources (41). From a cardiovascular nursing perspective, further evaluation of different tools across broader older adults with CVD groups is still needed to strengthen their use in nursing practice.

Patients in a state of pre-frailty may benefit from early, targeted interventions to prevent progression. Given the dynamic nature of frailty, reassessment during follow-up could help detect changes and adapt treatment. Nurses play a key role in screening for frailty in hospital settings. They are ideally positioned to identify patients at risk, coordinate transitional care, and tailor follow-up strategies to individual profiles.

In the post-COVID context, where healthcare delivery has been shaped by remote consultations and altered admission thresholds, structured frailty assessment may help maintain continuity of care and mitigate avoidable healthcare service use.

### 4.1 Limitations

This single-center study in a Spanish tertiary cardiology unit may limit generalizability, although the sample reflects older patients typically seen in European specialist care (9). The exclusion of patients with hospital stays shorter than three days may have introduced selection bias, potentially limiting the applicability of the findings to patients with shorter o less complex hospitalizations.

The low mortality rate (*n* = 2) reduced statistical power for survival analysis, but this was not a primary endpoint.

Frailty was measured with the translation of the Spanish FRAIL scale before formal validation (FRAIL-Es), yet semantic and structural integrity were preserved, and later studies support its use (42). The tool omits cognitive and psychosocial domains, possibly underestimating risk.

Post-discharge data came from regional public healthcare electronic records, so care outside the network may be missing, although follow-up losses were minimal. Assessment occurred within 72 h of admission, which may not capture changes during the stay, which may have been associated by acute illness and clinical instability, potentially introducing misclassification bias and not fully reflecting baseline status at discharge.

Given the exploratory nature of multivariable analyses and the limited number of events for some outcomes, results should be interpreted with caution.

Despite these limitations, the use of time-to-event analyses across multiple post-discharge outcomes provides insight into real-world healthcare trajectories in older adults with CVD.

## 5 Conclusions

This study supports the use of frailty-based stratification as a pragmatic approach to identifying older adults at risk of increased healthcare needs after hospital discharge. Frailty was associated with earlier, more frequent use of healthcare after discharge, particularly for non-cardiac care, reflecting vulnerability beyond the cardiovascular condition that led to hospital admission.

Frailty occurred alongside functional dependence, comorbidity, and markers of nutritional vulnerability. Simple indicators such as the Barthel Index, hemoglobin levels and low cholesterol may complement frailty assessment and aid in discharge planning.

The results support the incorporation of FRAIL scale into multidimensional assessment frameworks. Given the dynamic nature of frailty, system assessment during hospitalization and follow-up may enable timely interventions and help reduce potentially avoidable healthcare use. Further research in needed to explore the long-term impact of frailty-based strategies in ageing populations with CVD.

## Data Availability

The datasets presented in this article are not readily available due to privacy or ethical restrictions. Requests to access the datasets should be directed to nrivas@saludcastillayleon.es.
