# Supplementary material for: Frailty identifies early and non-cardiac healthcare utilization after cardiovascular hospitalization
Source: Front Cardiovasc Med. 2026 May 29;13:1806271. doi: 10.3389/fcvm.2026.1806271 (PMC13259912; doi:10.3389/fcvm.2026.1806271)
Supplement: Supplementary file 1 [file Table1.docx]

Supplementary Material

# Supplementary Tables

# Table S1. Association between time-to-events and frail vs. pre-frail and frail vs. robust statuses.

| Survival Outcome (Time-to-event: days) | Frailty Status Comparison | Mantel–Cox  χ² | df | p-value |
| --- | --- | --- | --- | --- |
| Time to first cardiology consultation | Frail vs. Robust | 2.78 | 1 | 0.096 |
|  | Frail vs. Pre-frail | 1.59 | 1 | 0.452 |
| Time to first non-cardiac consultation | Frail vs. Robust | 8.71 | 1 | 0.003** |
|  | Frail vs. Pre-frail | 2.72 | 1 | 0.099 |
| Time to emergency care | Frail vs. Robust | 2.08 | 1 | 0.149 |
|  | Frail vs. Pre-frail | 2.43 | 1 | 0.119 |
| Time to unplanned hospital readmission | Frail vs. Robust | 1.49 | 1 | 0.221 |
|  | Frail vs. Pre-frail | 5.01 | 1 | 0.025 * |
| Death | Frail vs. Robust | 0.652 | 1 | 0.419 |
|  | Frail vs. Pre-frail | 0.011 | 1 | 0.915 |

# Note: *p<0.05; **p<0.01.
